# Evaluation on Temperature-Dependent Transient *V*_T_ Instability in p-GaN Gate HEMTs under Negative Gate Stress by Fast Sweeping Characterization

**DOI:** 10.3390/mi13071096

**Published:** 2022-07-12

**Authors:** Rui Wang, Hui Guo, Qianyu Hou, Jianming Lei, Jin Wang, Junjun Xue, Bin Liu, Dunjun Chen, Hai Lu, Rong Zhang, Youdou Zheng

**Affiliations:** 1 Jiangsu Provincial Key Laboratory of Advanced Photonic and Electronic Materials, School of Electronic Science and Engineering, Nanjing University, Nanjing 210093, China; wrui@smail.nju.edu.cn (R.W.); guoh15@lzu.edu.cn (H.G.); houqianyu@smail.nju.edu.cn (Q.H.); bliu@nju.edu.cn (B.L.); hailu@nju.edu.cn (H.L.); rzhang@nju.edu.cn (R.Z.); ydzheng@nju.edu.cn (Y.Z.); 2 School of Electrical Engineering, Nanjing Vocational University of Industry Technology, Nanjing 210023, China; sumklee@163.com; 3 College of Electronic and Optical Engineering, Nanjing University of Posts and Telecommunications, Nanjing 210023, China; jin@njupt.edu.cn (J.W.); jjxue@njupt.edu.cn (J.X.)

**Keywords:** p-GaN gate AlGaN/GaN HEMT, NBTI, fast sweeping, threshold instability

## Abstract

In this work, temperature-dependent transient threshold voltage (*V*_T_) instability behaviors in p-GaN/AlGaN/GaN HEMTs, with both Schottky gate (SG) and Ohmic gate (OG), were investigated systematically, under negative gate bias stress, by a fast voltage sweeping method. For SG devices, a concave-shaped *V*_T_ evolution gradually occurs with the increase in temperature, and the concave peak appears faster with increasing reverse bias stress, followed by a corresponding convex-shaped *V*_T_ recovery process. In contrast, the concave-shaped *V*_T_ evolution for OG devices that occurred at room temperature gradually disappears in the opposite shifting direction with the increasing temperature, but the corresponding convex-shaped *V*_T_ recovery process is not observed, substituted, instead, with a quick and monotonic recovery process to the initial state. To explain these interesting and different phenomena, we proposed physical mechanisms of time and temperature-dependent hole trapping, releasing, and transport, in terms of the discrepancies in barrier height and space charge region, at the metal/p-GaN junction between SG and OG HEMTs.

## 1. Introduction

With superior performances of fast switching and low switching loss over Si/SiC counterparts, p-GaN gate AlGaN/GaN HEMTs are playing an increasingly important role in today’s power electronics market [1,2,3]. Under this background of accelerated industrial application, extensive studies are underway to understand and address the reliability concerns of devices [4,5].

The gate bias stress-induced threshold voltage (*V*_T_) instability of p-GaN gate HEMTs has been widely investigated recently, and the imbalanced extra charge accumulation, caused by the (de-) trapping of holes or electrons in the gate stack region, has been proposed as the main physical cause [6,7,8,9,10,11,12,13,14,15,16,17,18,19]. Considering that normally-off p-GaN gate devices feature a relatively low *V*_T_ voltage, applying negative gate voltage is an important method for improving the dv/dt robustness and preventing possible false turning-on induced by system noise [20,21,22]. Nevertheless, most studies have focused on the impacts of positive gate bias stress [6,7,8,9,10,11,12,13,14,15], but less attention has been given to the impact of negative gate bias stress on device *V*_T_ instability [4,16,17,18]. Elangovan et al. recently analyzed the instability behavior of Schottky-type E-mode p-GaN gate power HEMTs through pulsed negative gate bias stress tests and attributed the positive threshold shift (Δ*V*_T_) to the hole deficiency in the p-GaN region [17]. Zhang et al. also observed positive Δ*V*_T_ in the transfer characteristics of Schottky-type devices after prolonging negative gate bias stress time from 1 min to 60 min, and they attributed this positive Δ*V*_T_ to hole release from donor-type traps at the p-GaN/AlGaN hetero-interface [18].

Although the general law about threshold voltage shift and recovery is similar, the observed phenomena and corresponding physical explanations are quite different—or even contradictory—in detail, due to the differences in device structure, process, and the testing method [7,8,12]. Recently, the measurement time and the positive gate bias history, in conventional static transfer characteristic measurements, were found to have a significant influence on the reading of *V*_T_ values in p-GaN HEMTs [12,13,19]. For these reasons, fast voltage sweeping characterizations were recommended and adopted as useful methods to precisely capture transient *V*_T_ changes at the −μs time scale [12,13].

In this work, we employed specifically designed fast voltage sweeping measurements, on both Schottky and Ohmic-type p-GaN gate devices, to study negative gate bias stress-related *V*_T_ instability issues. Pulse-biased transfer tests and Measurement–Stress-Measurement (MSM) sequences were conducted to monitor the Δ*V*_T_ behaviors. From room temperature to elevated temperatures up to 150 °C, significant differences in transient Δ*V*_T_ evolution were captured, for both types of devices, over a wide time window, ranging from −µs to −ks during the stress and recovery processes in MSM sequences. These distinct phenomena offer a glimpse into more dynamic details, and the physical mechanisms are further analyzed accordingly.

## 2. Device Descriptions and Test Schemes

**Figure 1:**
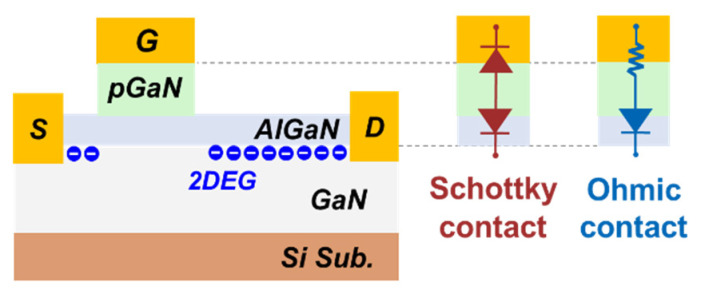
Schematics of the GaN HEMT device and gate structures.

The p-GaN gate HEMTs adopted in this work are commercially available devices with two different gate contact technologies, as shown in Figure 1. The Schottky-type gate (SG) p-GaN HEMT consists of two diodes connected back-to-back in the gate region: one is the metal/p-GaN junction, and the other one is the AlGaN/GaN interface. In this case, it features a reduced gate leakage current limited by the Schottky diode [23]. The other one is labeled as the Ohmic-type gate (OG) p-GaN HEMT that exhibits a higher gate leakage current, which could be modeled by a resistor and a diode connected in a series [24].

**Figure 2:**
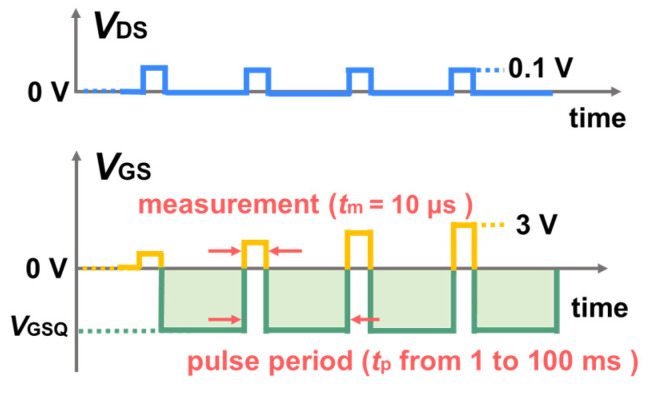
Schematics of a pulsed transfer characteristic test with negative quiescent gate bias (V_GSQ_).

Pulsed transfer characteristic tests with prolonged negative quiescent gate bias (*V*_GSQ_) were carried out under a Keithley 4200-SCS system with ultrafast I-V modules (4225-PMU). As shown in Figure 2, to minimize the extra charge accumulation introduced by the measurement [13], the pulse width (*t*_m_) of each test point was fixed at 10 μs, and the gate voltage scanning range was limited from 0 V to 3 V. The voltage step was 0.03 V, and 100 test points were adopted in each pulsed transfer measurement. Meanwhile, the pulse period was much longer, and it was set to no less than 1 ms, corresponding to a duty ratio of 1% or less. In addition, a time interval of 300 s was adopted between the adjacent pulsed transfer measurements to ensure a full recovery of *V*_T_ before each measurement.

**Figure 3:**
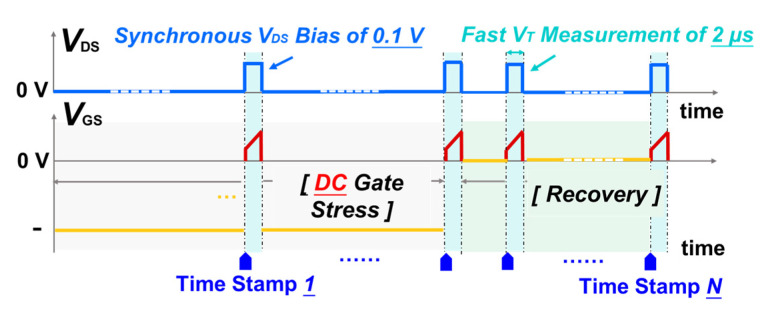
Schematics of an MSM sequence for transient Δ*V*_T_ characterization in stress and recovery processes under negative DC gate stress.

Although the prolonged pulse test method could roughly reflect the impact of negative gate bias stress and the stress duration on device *V*_T_ stability, it still lacks transient *V*_T_ shift (Δ*V*_T_) details in the stress and recovery processes. Thus, a series of MSM sequences with a −µs level of periodically inserted *I*_D_-*V*_G_ measurements were conducted to further explore the transient details, as shown in Figure 3. To minimize the testing error induced by extra charge accumulation, low forward gate sweeping voltages, from 1 V to 3 V and 0 V to 2 V, were applied on SG and OG HEMTs, respectively, in a short testing time of 2 µs. The influence of the sweeping measurements has been carefully pre-evaluated through without-stress monitory tests. In addition, a DC gate bias, varying from −4 V to −10 V, was adopted in the stress process, followed by a 1000 s recovery process with zero gate bias. Furthermore, the temperature-dependent transient Δ*V*_T_, from room temperature to 150 °C, was also evaluated under negative gate bias stress.

## 3. Results and Discussion

### 3.1. V_T_ Shift in Pulse Transfer Tests

**Figure 4:**
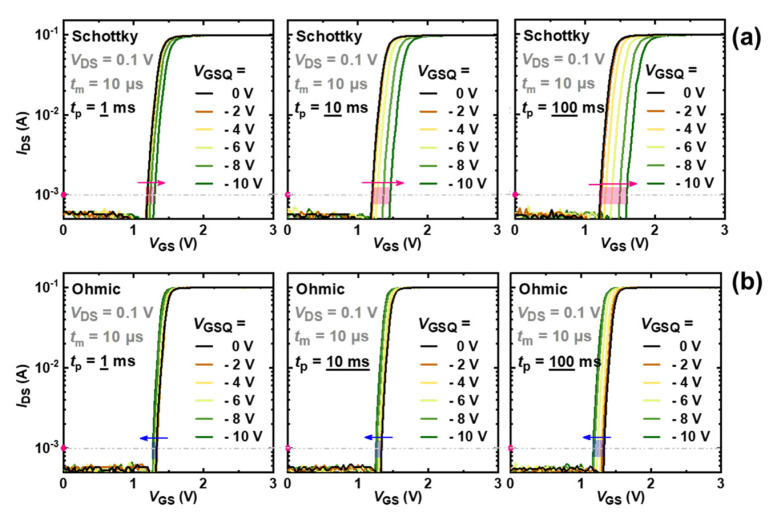
Pulsed transfer characteristic curves with *V*_GSQ_, from 0 V to −10 V, and pulse periods of 1 ms, 10 ms, and 100 ms: (**a**) SG HEMT, (**b**) OG HEMT.

As shown in Figure 4, the *V*_T_ shift of both two types of devices increases with increasing pulse stress time and negative gate stress bias, but their shift directions are opposite. For SG devices, *V*_T_ under *t*_p_ = 100 ms shows a forward shift, from approximately 1.2 V to 1.6 V, when *V*_GSQ_ varies from 0 V to −10 V, as shown in Figure 4a. In contrast, a smaller backward *V*_T_ shift, from around 1.30 to 1.25, is observed for OG devices under the same test conditions, as shown in Figure 4b. The different *V*_T_ shift behaviors indicate different extra charge accumulations in the gate region, and the detailed physical mechanism for this discrepancy will be further investigated by a series of MSM sequences.

### 3.2. Transient ΔV_T_ Evolution in MSM Sequences

**Figure 5:**
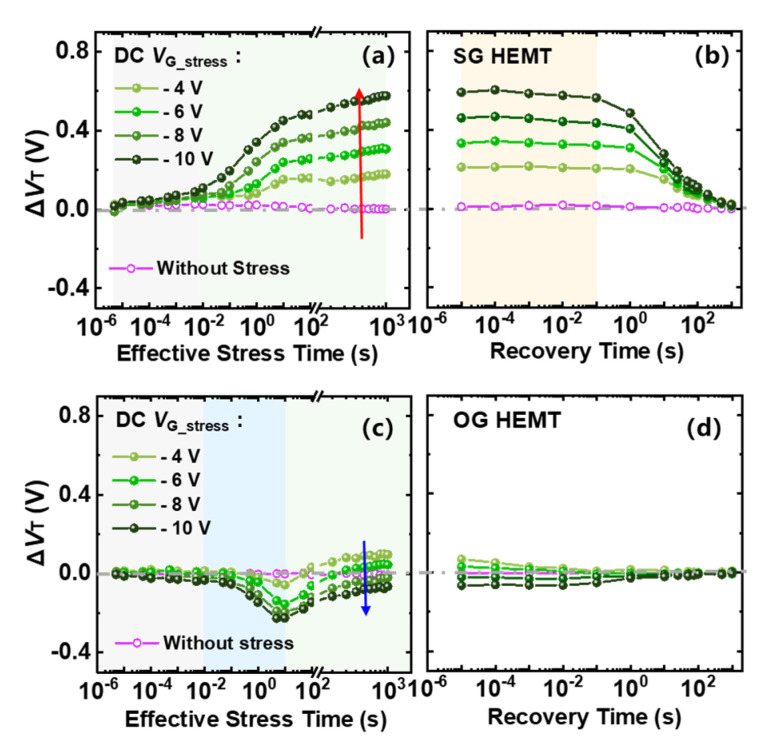
Transient Δ*V*_T_ evolution in 1000 s stress and subsequent 1000 s recovery processes, with gate stress from −4 V to −10 V, under room temperature: (**a**,**b**) SG HEMT, (**c**,**d**) OG HEMT.

As shown in Figure 5, the transient Δ*V*_T_ of SG HEMTs shows no obvious change during the initial stress process (5 µs–10 ms). As the stress time continues, Δ*V*_T_ begins to increase and becomes gradually saturated with a further increase in stress time. It is also worth mentioning that Δ*V*_T_ becomes more positive with increasing negative gate bias stress, as marked by the red arrow in Figure 5a. During the recovery process, as shown in Figure 5b, Δ*V*_T_ remains almost unchanged for the initial 0.1 s, and then, it can gradually recover to the initial state in the subsequent 1000 s. Compared with SG HEMTs, OG HEMTs feature a smaller amplitude in Δ*V*_T_ under the same stress conditions. In addition, Δ*V*_T_ shows non-monotonic change as the stress time increases. More specifically, Δ*V*_T_ first drops to a negative value, after approximately 1 s of stress time, as marked by the blue shade in Figure 5c. After that, Δ*V*_T_ gradually rises to a stable state. Besides, the change trend of Δ*V*_T_, with the gate bias stress in OG HEMTs, is opposite to that in SG HEMTs, as marked by the blue arrow in Figure 5c. In the subsequent recovery process, OG HEMTs demonstrate a faster recovery speed, where *V*_T_ starts the recovery process immediately after the removal of gate bias stress and can fully recover to the initial state in approximately 1 s recovery time.

**Figure 6:**
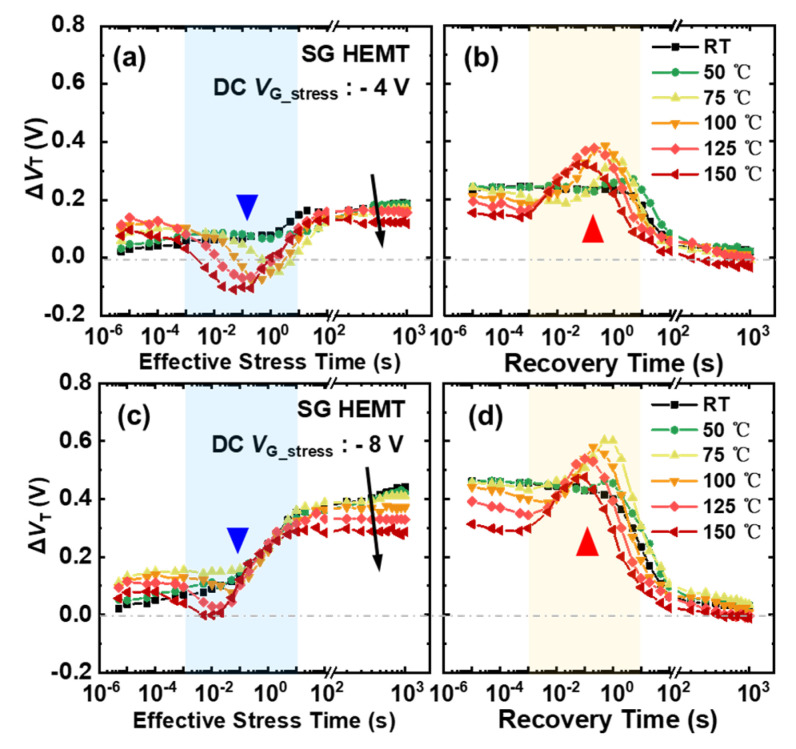
Δ*V*_T_ evolution of SG HEMT in 1000 s stress and subsequent 1000 s recovery processes under elevated temperature, with gate bias stress at: (**a**,**b**) −4 V and (**c**,**d**) −8 V.

However, the Δ*V*_T_ evolution, with stress and recovery time, at elevated temperatures is found to be very different from the behavior at room temperature, for both SG and OG HEMTs, when conducting negative bias stress MSM sequences. As shown in Figure 6, Δ*V*_T_, with stress time in SG HEMTs, features a concave-shaped evolution process under the given negative bias stress, and the concave shape occurs earlier and features a larger amplitude with the increasing temperature. It is very interesting that a convex-shaped *V*_T_ evolution appears, correspondingly, in the recovery process. In addition, as marked by the black arrows, the final saturated Δ*V*_T_ decreases with the increasing temperature under the given gate bias stress.

**Figure 7:**
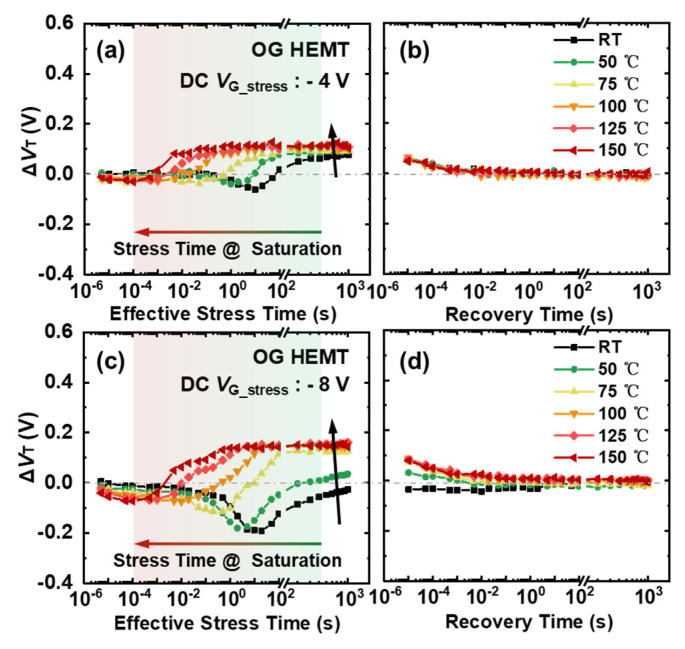
Δ*V*_T_ evolution of OG HEMT in 1000 s stress and subsequent 1000 s recovery processes, under elevated temperature, with gate bias stress at: (**a**,**b**) −4 V and (**c**,**d**) −8 V.

For OG HEMTs, the change in Δ*V*_T_ is generally smaller in comparison with SG HEMTs. As shown in Figure 7a–c, Δ*V*_T_ becomes slightly more negative in the initial stress process and, then, begins to rise to a stable and positive value earlier, with the increasing temperature, for all the given gate bias stress. The time to reach the stable state for Δ*V*_T_ is greatly shortened from 100 s to 1 ms when the temperature varies from room temperature to 150 °C. In addition, this stable Δ*V*_T_ seems to grow gradually saturated with further increases in temperature. The recovery process becomes simpler with a quick and monotonous change to the initial state.

### 3.3. Physical Mechanism Analysis

**Figure 8:**
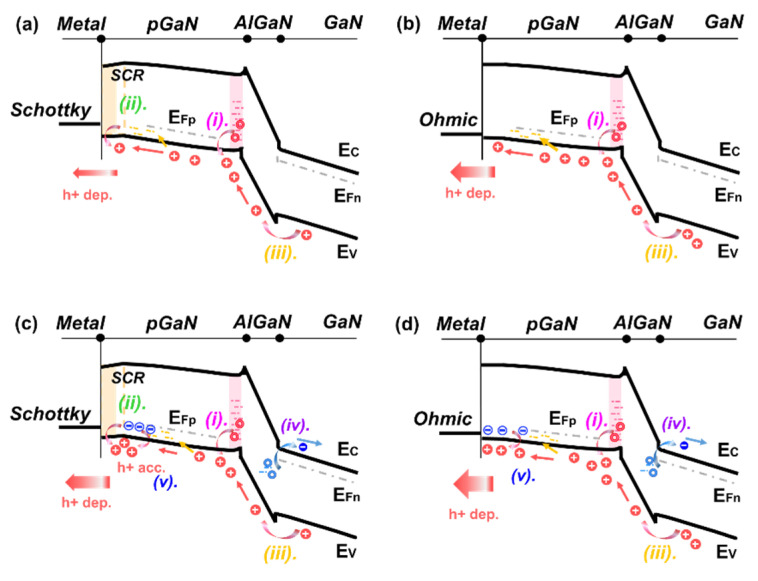
Schematic band diagrams of the gate stack region in the negative gate stress process under room temperature: (**a**) SG HEMT, (**b**) OG HEMT; under elevated temperature: (**c**) SG HEMT, (**d**) OG HEMT.

The transient Δ*V*_T_ evolution of devices during stress and subsequent recovery processes, presented above, reflects the dynamic changes of net charges underneath the gate stack region. Figure 8 illustrates the schematic energy band diagram of the device gate stack region under negative gate bias stress. Under the stress condition, the p-GaN/AlGaN/GaN heterojunction is reverse biased, and a depletion region takes place, as marked by the red shadow. In particular, the metal/p-GaN Schottky junction in SG HEMTs is forward biased, and it features an additional Space Charge Region (SCR) compared to that in OG HEMTs, as marked by the yellow shadow.

At room temperature, little electron injection or a trapping process could be introduced under the negative gate bias stress [18], while the depletion or accumulation of holes plays a major role and is further investigated. The positive Δ*V*_T_ during the gate stress process could stem from the hole emission of the gate-stack region of p-GaN gate HEMTs, which is equivalent to the depletion of net positive charges. In contrast, the negative Δ*V*_T_ results from hole accumulation. The behavior of Δ*V*_T_ could be linked to three physical processes, as illustrated in Figure 8a,b:

1. Donor-type hole trap states at the p-GaN/AlGaN interface could be activated and release holes to the valence band in the p-GaN layer [17,18];
2. The depletion width of SCR, in the p-GaN layer of SG HEMTs, would decrease under the negative gate bias stress, which also leads to hole release [4];
3. Holes could flow from the gate-source drift region, towards the gate stack, and under large negative gate bias stress. Part of the holes may flow out to the gate terminal and contribute to the gate current, while part of the holes may get trapped into the gate stack region and lead to an extra hole accumulation [16].

**Figure 9:**
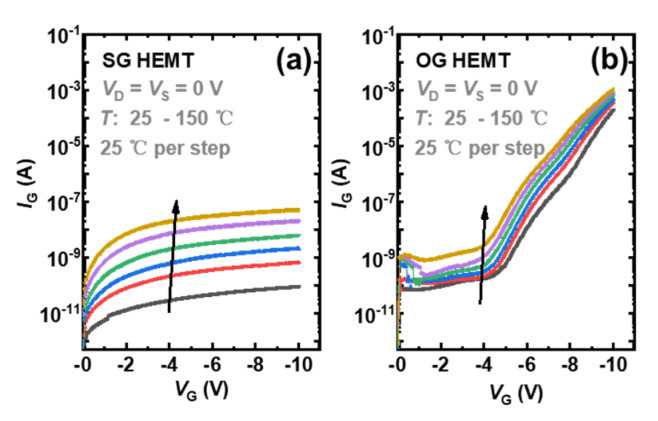
Gate leakage current of p-GaN gate devices under various temperatures: (**a**) SG HEMT, (**b**) OG HEMT.

In SG HEMTs, the increase in the saturated Δ*V*_T_, with increasing negative bias stress in Figure 5a, corresponds to further depletion of holes. With the increasing electric field under higher bias stress, more holes released in process (i)–(iii) could be emitted to gate metal and drift away, leading to a higher extent of hole deficiency. In contrast, OG HEMTs exhibit a smaller *V*_T_ shift toward the opposite direction with increasing negative bias stress, as shown in Figure 5c. For OG HEMTs, holes would be less released through process (ii) because of the absence of the SCR. In addition, the p-i-n diode mainly undertakes the reverse-bias voltage, and the electric field drop across the AlGaN barrier is larger in OG HEMTs, compared with that in SG HEMTs, under given negative gate stress. Thus, it is easier for a hole to cross the AlGaN barrier layer and inject into the gate stack region in OG HEMT. Besides, with an increasing negative bias, OG HEMTs feature a larger gate leakage current, which is six orders of magnitude higher than that in SG HEMTs, under a given gate bias of −10 V, as shown in Figure 9. The increased difference in the gate leakage current, under large reverse gate bias, indicates that the process (iii) plays an important role in OG HEMTs. As a result, hole accumulation gradually becomes dominated in the gate stack region of OG HEMTs, as negative gate bias stress increases and, hence, leads to a more negative Δ*V*_T_. Moreover, in the subsequent recovery processes, SG HEMTs demonstrate a slower recovery process compared to OG HEMTs Figure 5b,d. This phenomenon could be attributed to the presence of the Schottky barrier at the metal/p-GaN junction in SG HEMTs. Different from OG HEMTs, the p-GaN bulk begins electrically floating with the formation of Schottky-type contacts at the metal/p-GaN interface in SG HEMTs. When the stress is withdrawn, the previously reduced SCR depletion width in the p-GaN layer of SG HEMTs is restored, and the valance–band offset at the metal/p-GaN junction increases and returns to the original state. This Schottky barrier will hinder the backflow of those holes depleted in the stress phase and, hence, results in a slower recovery process in SG HEMTs.

In principle, both hole injection or release in processes (i)–(iii) could be enhanced through thermal assistance under elevated temperatures. Additionally, more detailed physical processes should be taken into consideration, as shown in Figure 8c,d:

1. The deep-level defects in the AlGaN layer could be activated with an increasing temperature and participate in the de-trapping process of electrons at the negative gate bias;
2. The acceptors in the p-GaN cap layer could be quickly ionized with an increasing temperature and, hence, release holes at the negative gate bias, leaving net negative charges behind.

As a result, the distinct transient Δ*V*_T_ behavior, under the elevated temperature for two types of devices, could be attributed to a combination of the above processes. For SG HEMTs, the concave-shaped Δ*V*_T_ evolution, in the range of around 10 ms to 1 s stress time, could be linked to a temporary accumulation of net positive charges during the initial stress stage. For one thing, the electrons released by the activated deep-level defects, through the process (iv), are driven away from the gate stack region under the electric field. Meanwhile, more holes could also be released through processes (v) with increasing temperatures, and these extra released holes could not fully drift away in short time, due to the block of Schottky barrier. Consequently, the more obvious concave-shaped Δ*V*_T_ evolution occurs with increasing temperatures.

In addition, the convex-shaped Δ*V*_T_ evolution in the recovery process is also associated with the involvement of process (iv). When the bias stress is withdrawn, the electrons driven away during the stress process gradually flow back to the gate stack region. However, the holes could not be replenished immediately, due to the blocking of the Schottky barrier. For these reasons, negative net charge accumulation becomes temporarily dominated, and hence, convex-shaped Δ*V*_T_ evolution occurs in the recovery process. As recovery time increases, the number of electrons re-captured by the deep-level defects becomes saturated. The transient Δ*V*_T_ could recover to the initial state as the reduced positive charges are gradually restored. Moreover, the reduction in the saturated Δ*V*_T_ with an increasing temperature could be ascribed to the participation of processes (v). With the increasing ionization of acceptors under elevated temperatures, more net negative charges are left behind and could balance the voltage drop on the gate stack region, hence resulting in a reduction in the saturated Δ*V*_T_ under the given gate bias stress.

For OG HEMTs, the concave-shaped *V*_T_ evolution that occurred at room temperature gradually disappears, and the time of reaching the saturated Δ*V*_T_ becomes shorter with the increasing temperature under the given gate bias stress. These phenomena indicate an accelerated hole depletion process underneath the gate stack region. With lower barrier height in OG HEMTs, more holes under elevated temperatures could be emitted to the gate metal and drift away. In addition, the recovery process in OG HEMTs is simpler and exhibits quick and monotonic change towards the initial state, which could also be attributed to the flowing of holes back under the low barrier being easier.

## 4. Conclusions

In summary, the temperature-dependent transient Δ*V*_T_ evolution in OG and SG HEMTs, under negative gate bias stress, are captured in the *−μs* to *−ks* time window by fast sweeping characterizations, and some interesting phenomena are observed. As the temperature increases, SG HEMTs exhibit distinct concave-shaped and convex-shaped Δ*V*_T_ evolution in the stress and recovery processes, separately, while OG HEMTs exhibit an accelerated Δ*V*_T_ increase, towards the positive direction, in the stress process and monotonic change in the recovery process. These transient details reflect the dynamic net charge variations underneath the gate stack region, and the time and temperature-dependent hole trapping, releasing, and transport processes are further analyzed through the proposed physical mechanisms. The comprehensive mechanism analysis takes account of the differences in device structures, as well as the participation of different physical processes under elevated temperatures, which contributes to a better understanding on the characteristics of p-GaN gate HEMTs.
